# Drunkenness

**Published:** 1872-07

**Authors:** 


### DRUNKENNESS.

NEARLY a hundred years ago, a New York lawyer was travelling on horseback on Long Island, and coming to an inviting country inn, at the close of the day, he alighted for the purpose of spending the night. The family consisted of the father, mother, and daughter of eighteen, so retired in her manners, so comely in her person, and of a mind so cultivated and refined, that on leaving next morning the young man determined that he would repeat his visit before a great while, which he did. In due time they were married, went to housekeeping in New York, and set about the business of life in real earnest. The young lawyer rose in his profession, made a name, lived happily, temperately, and long, dying at a good old age, leaving a large fortune to two surviving sons, one of whom died within easy memory of the New York Knickerbocker, a besotted, driveling drunkard, leaving two sons, both of whom

"TOOK TO DRINK"

early, and early died childless. The other son of the lawyer married a beautiful and accomplished woman, himself a handsome man, of refinement and culture; but he fell into drinking habits, spent his own patrimony, and a large share of his wife's fortune. His infirmity grew upon him to an extent which made it impossible for his wife to live with him longer, and, taking her grown daughter with her, she left him, legally married again, and is now living happily with the second man of her choice, and another family of children growing up around her; all these things made such a profound impression on the mind of the forsaken father and husband, that he resolved he would never drink another drop of liquor again, and for twenty years has kept his resolution.

Was this turning to drink on the part of the children and grandchildren the result of enticements into bad habits, or by inheritance? The lawyer and his wife were strictly temperate in all their habits, and plain in their tastes and modes of living, but cultivated, hospitable, and refined. When the keeper of the Long Island country inn married, he was a well-to-do young man, fond of his wife, and fond of his home; but his occupation, particularly at that early day, led very naturally into habits of drinking; it was a common thing then to

"TREAT"

friends to a drink of grog when they happened to drop in during the day, and when night came, the neighbors would come in to learn the news from the city, as gathered from passing travellers; and this easily degenerated into sipping toddy and brandy and water during the evening chat, which by degrees extended into the night; the landlord, it is reported, generally going to bed full of liquor, rousing the "strong propensities" of nature, which would not be quieted without the fullest gratification. Under these influences a new being was made. But the leprosy of drunkenness did not break out in the first generation; the habits, the cultivation, and the refinement of the inn-keeper's daughter were all in antagonism to what might foster the habit of drink, and so it skipped over a generation, the tinder being applied to the torch to be kindled into flame under the greater susceptibilities of boyhood life and surroundings. It is precisely in this way that

INSANITY

overleaps a generation or two; thus also it is that a child bears no resemblance to its father or mother, but is often the exact resemblance to the grandparents or great-grandparents. Doubtless, in innumerable cases, the foundation of drunkenness in persons yet unborn has been laid by parents retiring after the sumptuous dinner, or the evening party, one or both saturated with wine, or worse. Let the terrible truth impress itself on the thoughtful reader's mind, that in a Massachusetts asylum for the cure of idiotic children, three fourths were born of parents one or both of whom were habitual drinkers of spirituous liquors. It is surely not necessary to state more clearly the inferences to be drawn from these observations, and yet men are so dull of comprehension sometimes as to require the plainest teachings; and still, this lesson is of importance but little less than infinite; it suggests the abeyance of perpetuative function when under alcoholic influence. The self-same lesson is powerfully taught in the facts recorded in standard medical works, showing that if a mother suckles her infant within half an hour after being in an ungovernable rage, it will be immediately thrown into convulsions. The great broad fact then remains, that mental and physical constitutions, appetites, propensities, and passions, which mould the physical condition of the infant nursed under their influences, fix the character of the being begotten at the time of their prevalence. And under this most important practical principle, having such a controlling power in forming the characters and fixing the destinies of the unborn, as well as the babe, are ranged that large class of what are regarded as

MYSTERIOUS CASES, where children are so totally different from their parents in their mental and moral characteristics. Nothing can so well account for the character of

AARON BURR, the first-born and only son of father and mother, models of humble piety, of Christian devotion, and of stern faith in Calvinistic doctrine — yet leaving that son, magnificent in his talents, but an infidel in religion, without moral principle, a roué, a traitor, and a murderer; the father or mother, or both, when he was begotten, laboring under those depressing influences of doubt and unbelief or temporary rebellion against the Divine government, which sometimes prevail for a transient period in the experiences of the wisest and best of men; for there were times in the lives of such as David and Knox and Chalmers and Cowper and Newton, when the sirocco of unbelief would sweep across their hearts, scorching up all that was

GREEN AND GOODLY

to look upon in moral and religious feeling and sentiment, tempting the mind to express itself in the words of the fool —

" THERE IS NO GOD."

